# Modelling arterial thrombus formation *in vitro*

**DOI:** 10.1097/MOH.0000000000000789

**Published:** 2023-11-03

**Authors:** Amelia Drysdale, Azziza Zaabalawi, Sarah Jones

**Affiliations:** Department of Life Sciences, Faculty of Science and Engineering, Manchester Metropolitan University, Manchester, UK

**Keywords:** endothelium, microfluidic, platelet, thrombosis

## Abstract

**Purpose of review:**

Models of arterial thrombus formation represent a vital experimental tool to investigate platelet function and test novel antithrombotic drugs. This review highlights some of the recent advances in modelling thrombus formation *in vitro* and suggests potential future directions.

**Recent findings:**

Microfluidic devices and the availability of commercial chips in addition to enhanced accessibility of 3D printing has facilitated a rapid surge in the development of novel in-vitro thrombosis models. These include progression towards more sophisticated, ‘vessel on a chip’ models which incorporate vascular endothelial cells and smooth muscle cells. Other approaches include the addition of branches to the traditional single channel to yield an occlusive model; and developments in the adhesive coating of microfluidic chambers to better mimic the thrombogenic surface exposed following plaque rupture. Future developments in the drive to create more biologically relevant chambers could see a move towards the use of human placental vessels, perfused ex-vivo. However, further work is required to determine the feasibility and validity of this approach.

**Summary:**

Recent advances in thrombus formation models have significantly improved the pathophysiological relevance of in-vitro flow chambers to better reflect the in-vivo environment and provide a more translational platform to test novel antithrombotics.

## INTRODUCTION

The use of experimental in-vivo and in-vitro thrombosis models has been fundamental in furthering our knowledge of arterial thrombosis, from understanding the mechanisms governing thrombosis through to the screening of novel antiplatelet drugs. The most common in-vivo models of arterial thrombosis are murine models where the vascular endothelium is damaged using laser ablation or chemically damaged using ferric chloride [1]. The size of the thrombus, or extent of vascular occlusion is then measured using intravital microscopy or Doppler ultrasound respectively [2]. The use of murine models has many advantages, in particular the ability to genetically modify platelets and the presence of the complete circulatory system [3]. However, the models lack standardization and there is considerable variability between different labs [1]. The relevance of murine models to human thrombosis must also be questioned, given the significant biochemical, anatomical, and physiological species differences. This may in part explain the poor translation from animal studies to clinic, with very few novel antithrombotics emerging over the last decade. Those which have emerged include Vorapaxar, which targets the protease-activated receptor 1 (PAR1) receptor not found on murine platelets [4] and Glenzocimab, a humanized monoclonal antibody fragment directed against the human platelet glycoprotein receptor (GPVI) [5]. Studies on C-type lectin receptor 2 (CLEC2) have further emphasized the importance of species differences with profound inhibition of arterial thrombosis demonstrated in CLEC2 deficient mouse platelets but no difference observed in thrombus formation with human platelets following CLEC2 inhibition [6^▪^]. The overreliance on murine thrombosis models may therefore be hindering the discovery of novel human antithrombotics. The acknowledgement of the need for humanized models, combined with recent advances in 3D printing, commercial chips and microfluidics has driven a surge in novel in-vitro thrombosis models which have the benefit of using human blood, human cells and shear rates representative of human coronary arteries. Here we provide a brief overview of the evolution of in-vitro thrombosis models and critically evaluate recent advances in the field. 

**Box 1:**
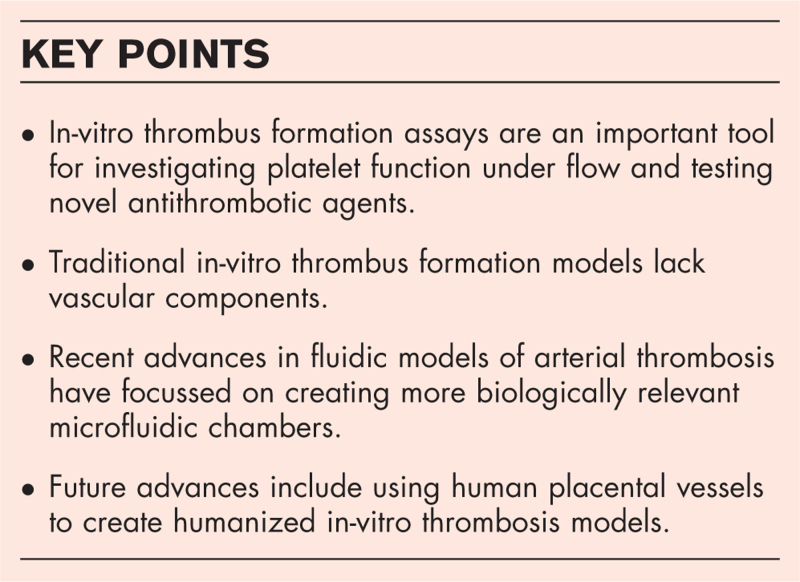
no caption available

## THE DEVELOPMENT OF IN-VITRO THROMBOSIS MODELS

**FIGURE 1:**
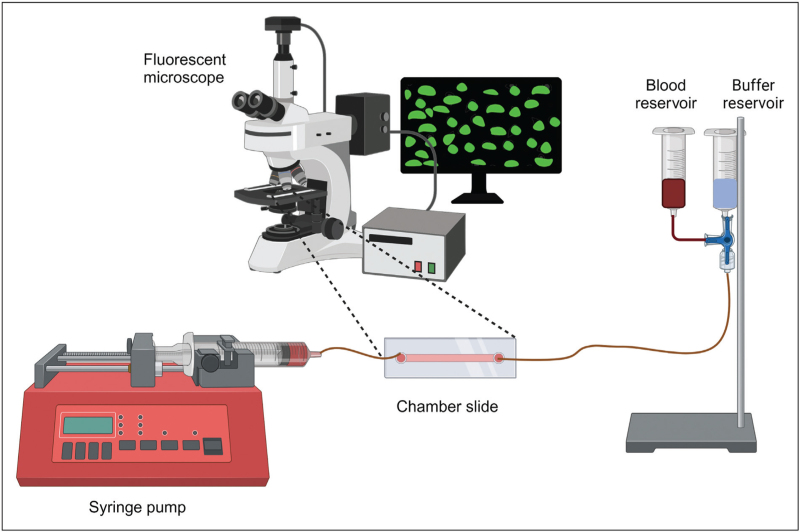
Traditional in-vitro thrombus formation model. The standard in-vitro thrombosis model routinely used to study platelet adhesion and thrombus formation under arterial flow conditions comprises a reservoir for whole blood and an additional reservoir for physiological buffer; a custom-made or commercial flow chamber usually coated with Type I fibrillar collagen and a syringe pump which draws blood from the reservoir through the flow chamber at a set flow rate to achieve a defined arterial shear rate. A fluorescent dye or antibody is usually added to the whole blood prior to the experiment to facilitate visualization of platelet adhesion and thrombus formation in real-time using fluorescence microscopy.

In-vitro thrombosis models have been around since the 1930s with one of the earliest models being the capillary thrombometer [7], which was instrumental in the early descriptions of von Willebrand's disease [7–9]. It comprised a glass capillary with glass columns either side, allowing bidirectional flow of blood through the capillary and thrombus time to be measured. Parallel plate flow systems followed in the 1970s, which enabled more controlled experiments, using defined unidirectional flow and chambers coated with extracellular matrix (ECM) proteins to measure platelet adhesion and thrombus formation under arterial flow conditions [10,11]. The popularity of these models increased over the following decades and evolved to allow more precise flow conditions and smaller blood volumes. The current basic in-vitro thrombosis model used routinely in the platelet field involves the perfusion of whole blood through single-channel parallel-plate flow chambers or microslides coated with Type 1 fibrillar collagen. Platelets are usually fluorescently labelled, allowing real-time analysis of platelet adhesion and thrombus formation, and the quantification of thrombus size (Fig. 1).

Type I collagen is the most thrombogenic extracellular matrix protein found in the blood vessel wall [12] and is enhanced in atherosclerotic lesions [13]. This simple in-vitro flow system therefore enables the early steps of arterial thrombus formation to be assessed including tethering of platelets to collagen indirectly via von Willebrand factor (VWF) and directly through platelet GPVI and α2β1 receptors; platelet activation and recruitment, through the release of soluble mediators; and platelet aggregation and thrombus growth [14]. The flexibility of the in-vitro system enables flow rates to be altered to mimic different vascular beds or pathological conditions, for example arterial stenosis, where wall shear stress is significantly elevated [15]. Chambers can also be coated with alternative adhesive ligands or combined with tissue factor (TF) to mimic the large amounts of TF exposed following the rupture of an advanced atherosclerotic plaque [16].

In-vitro thrombus formation assays have proved pivotal in characterizing the mechanism by which VWF and the GPIb-IX-V complex facilitates the initial platelet tethering to collagen [17,18], the importance of GPVI [19–21] and the central role of integrin αIIbβ3 [18]. They have also provided an important platform for the identification of numerous novel modulators of thrombus growth and stability [22–27]. Despite the advantages offered by in-vitro thrombosis models, there remain several limitations which researchers have attempted to address over recent years. Firstly, the internal environment of the flow chambers fails to mimic the local environment where arterial thrombi usually form. The absence of cellular components, lack of a complex cell derived ECM, and the rigidity of the flow chambers are all factors which limit the translation of traditional flow models.

Secondly, the linear kinetics of thrombus growth observed in flow chambers is different to the dynamic thrombus assembly observed in mouse models, where disaggregation and embolization occur prior to thrombus stabilization [5,28]. It is unclear whether this is a species-specific phenomenon, or whether this is due to the lack of endothelial cells and the negative regulation that they provide. Advances to include endothelial cells in in-vitro thrombosis models may address this issue. Another limitation is the failure of current models to achieve occlusion. This is likely due to the single channel set-up where the lack of collateral vessels results in excessive pump pressure dislodging the thrombi that have formed.

Finally, the single channel flow models are relatively low throughput impeding their uptake commercially to screen for novel antithrombotic compounds, and clinically to assess thrombus formation on a range of adhesive ligands. In this review we discuss the most important recent advances and approaches, which address many of the limitations of the traditional in-vitro thrombosis model.

## ADVANCES IN MICROFLUIDICS

Microfluidic chambers enable investigations demonstrating the influence of whole blood rheology on platelet activity and thrombus formation, as they often use smaller amounts of blood, increasing accessibility [29]. Additionally, these models reduce time, cost and ethical issues surrounding current in-vivo models [30]. Popular custom-made microfluidic devices are biocompatible polydimethylsiloxane (PDMS)-based, and incorporate well plates [31] and, more traditionally, chambers [32]. These devices have been used to investigate platelet behaviour under different conditions, on various substrates and under varying shear rates, and are inexpensive, however lack of standardization of these custom-made devices limits widespread uptake [33]. Over the last decade, a variety of commercially available microslides and biochips have emerged facilitating standardization and more widespread use [27]. Recent advances in the use of both PDMS-based devices and commercial chamber slides are summarised below.

## HIGH-THROUGHPUT IN-VITRO MODELS

The early development of in-vitro models consisted of whole blood perfused over Type I Horm collagen, sometimes in combination with tissue factor [34], to investigate the efficacy of antiplatelet therapy under different conditions [35] as well as pathologies such as von Willebrand disease, haemophilia and various platelet disorders [36]. Experimental design, however, meant that only single channels could be perfused at one time, limiting the number of experiments that could be performed with fresh blood. Advances in commercial microfluidic devices, with chips containing multiple channels, as well as pumps allowing simultaneous perfusion of 6–8 channels has significantly improved the efficiency of these models.

An alternative approach which has also enhanced the throughput of in-vitro flow experiments is microspotting pioneered by Cosemans and Heemskerk [37–40]. Precise microspotting of an array of extracellular matrix proteins on a coverslip, prior to assembly in a parallel flow chamber, facilitates multiple parameters to be assessed simultaneously [37]. A potential limitation of this model is priming of platelets as they flow over one spot, therefore affecting responses to downstream spots, however control experiments demonstrated no differences in thrombus formation based on the order of coated proteins [37]. The flexibility of this model has resulted in its extensive use over the past few years in a diverse range of experiments including assessment of variations in thrombus formation between healthy individuals [39], assessment of coagulation under flow [41,42], phenotyping patients with platelet disorders [40,43^▪^], assessment of pharmacological GPVI and integrin α2β1 inhibitors [44], characterization of calcium entry pathways in platelets [45] and assessment of the platelet inhibitory effects of different cancer treatments [46].

## PLAQUE COMPONENTS AND CELL DERIVED MATRICES

Arterial thrombosis is predominantly triggered by the rupture or erosion of an atherosclerotic plaque [47]. To recapitulate the in-vivo vessel environment and the rupture of a vulnerable atherosclerotic plaque, tissue factor, in combination with fibrillar collagen is used in in-vitro thrombosis models to trigger the coagulation cascade. However, other plaque components are present at the plaque-thrombus interface, including other extracellular matrix proteins [47], necrotic core, endothelial cells and vascular smooth muscle cells. To increase the biological relevance of in-vitro models, these components must be considered as contributors to the formation of arterial thrombi and may also influence antithrombotic efficacy. One adaptation to the basic thrombosis model, to specifically address this issue is the use of atherosclerotic plaque homogenates, to provide the thrombogenic surface in flow chambers [48,49]. Although not routinely used, plaque homogenates have been used to investigate current and novel antithrombotic agents [25,50]. Challenges do exist in achieving a stable, consistent coating, with heterogeneity in collagen content demonstrated [49]. Disparities in coating coverslips with plaque homogenate have recently been addressed by Karel *et al.*[48], who spin-coated coverslips, achieving coating consistency and enabling replicable investigations using this material [51]. However, this does not overcome the problem of considerable plaque heterogeneity between individuals.

The isolation of plaque material from carotid endarterectomy and the homogenisation process, may affect the structural arrangement of the different extracellular matrix proteins, altering the function compared to the atherosclerotic lesion *in situ*. A lack of fibrillar collagen has been reported in homogenates [49], which may in part explain recent findings demonstrating differential GPVI activation on fibrillar collagen compared to plaque homogenate [48,52]. Our own studies, however, have also demonstrated a significant difference in thrombus formation on Type 1 Horm collagen compared to native extracellular matrix proteins produced by human coronary artery endothelial cells (Drysdale and Jones, unpublished data). This suggests that Horm collagen isolated from horse tendon is significantly more thrombogenic than vascular Type 1 collagen, or that other components of the native matrix, such as proteoglycans negatively regulate platelet responses to collagen [47].

## ENDOTHELIAL CONTRIBUTIONS

The use of plaque material within in-vitro thrombosis models provides a trigger for arterial thrombosis much more akin to the in-vivo scenario; however, these advances are still limited by the lack of vascular cells, in particular endothelial cells, and their important contributions to arterial thrombus formation. Indeed, endothelial cells are key regulators of platelets *in vivo,* with endothelial dysfunction an important promoter of platelet activation and aberrant thrombus formation [53,54]. Several studies have recently emerged, where endothelialized chambers have been developed to assess thrombus formation under arterial flow conditions. Brouns *et al.*[41] cultured a sub-confluent layer of human umbilical vein endothelial cells (HUVECs) on Type I collagen and tissue factor to investigate thrombus formation. They demonstrated that the presence of HUVECs delayed platelet adhesion and fibrin formation, which was reversed by the addition of heparinase III [41]. Although the model demonstrates an inhibitory effect on thrombus formation by the endothelial cells, the model is limited by the lack of a confluent endothelial monolayer and a lack of endothelial damage or dysfunction, which is usually observed in patients with cardiovascular disease. Banka and Eniola-Adefeso [55^▪▪^], overcame some of these limitations by culturing HUVECs to confluency on collagen coated coverslips and using a scalpel to create a scratch exposing the collagen, in addition to acute activation of the cells with histamine. The study demonstrated enhanced platelet adhesion evoked by histamine induced VWF release and also provided proof of principal that the model could be used to assess antithrombotic drugs which target the endothelium [55^▪▪^].

A simple but effective approach that we have developed to evaluate endothelial contributions to thrombus formation and antithrombotic efficacy is to connect two adjacent microfluidic chambers, the first containing a confluent monolayer of HCAECs and the second type I collagen. This offers the advantage of disease relevant endothelial cells and the ability to use commercially available chips. The disadvantage however is that direct effects of the endothelial cells cannot be measured, only the effects of soluble mediators released from the cells such as nitric oxide and prostacyclin. Using this model, we demonstrated that healthy HCAECs significantly reduced thrombus formation, which was abolished in dysfunctional HCAECs largely through the loss of eNOS (Riley *et al.*, unpublished data).

A further limitation of the endothelialized models described in the literature thus far is the absence of flow conditioning. It is well established that haemodynamics significantly alters the biology of endothelial cells, with laminar flow being atheroprotective and disturbed flow evoking an atheroprone phenotype [56]. Future advances in endothelialized models should consider the importance of shear stress, however the benefits of incorporating flow conditioning must be balanced against the associated challenges of doing so. Cells would require flow conditioning for a minimum of 72 h to achieve a stable phenotype [57] and specialist closed system fluidic pumps are required to maintain long term perfusion, adding significant time and cost to experimental protocols.

## OTHER VASCULAR COMPONENTS AND ARTERIAL CONSTRUCTS

Advances towards more vessel like in-vitro models have now progressed beyond the inclusion of endothelial cells to vascular constructs in an attempt to include other vascular components and avoid the use of rigid chambers which are much stiffer than the vascular wall [58^▪▪^]. To achieve this, bioprinting has been adopted. Briefly, a pattern representing a vascular network is created and cast by a hydrogel (usually Type I collagen-based), before being removed, leaving behind channels which can be seeded with endothelial cells and perfused with cell culture medium or whole blood [59]. These models are reproducible and can be used to investigate cell behaviour, co-culture crosstalk and transendothelial migration [60]. Recent advances in the use of hydrogels include the development of biomimetic constructs of the medial and adventitial layers of the vascular wall containing smooth muscle cells and fibroblasts [61], providing a more representative substrate for endothelial cell seeding, compared with Type I collagen. Additionally, hydrogel-based designs have been used to model atherothrombosis, using varying degrees of vessel stenosis [62]. The disadvantage of hydrogel-based systems, however, is the reliance on the use of custom-made moulds and expertise to build and operate, discouraging widespread uptake by researchers. Additionally, it is difficult to perform thrombus formation investigations in these models, as there is often no methodology to damage endothelial cells and expose the underlying thrombogenic extracellular matrix. Indeed, this is a factor limiting the use of cells in current modelling systems.

## OCCLUSIVE IN-VITRO THROMBOSIS MODELS

Historically, in-vitro thrombosis models are largely nonocclusive, due to the dislodgement of large thrombi in chambers caused by the build-up in pressure and the lack of co-lateral vessels providing an alternative route of less resistance. Recently, however a series of adaptations to the standard flow chamber model has enabled an occlusive model of thrombosis to be established [63^▪▪^]. Berry *et al.*[63^▪▪^] created a custom-made pressure relief PDMS-based model to generate occlusive clot formation on a patch coating of Type I Horm collagen and tissue factor, enabling measurement of time to occlusion following treatment with antiplatelet drugs. The model was successful in occluding chambers due to the incorporation of a branching design, with blood flow able to divert following a build-up of fluid pressure. However, off-site coagulation occurring downstream of the patch was observed and required downstream infusion of ethylenediaminetetraacetic acid (EDTA) [63^▪▪^], potentially due to the absence of endothelial cell regulation in the model.

## FUTURE PERSPECTIVES

**FIGURE 2:**
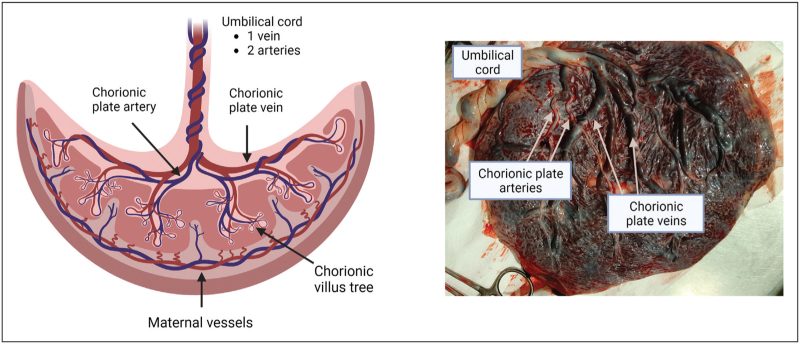
Human placental vessels. (a) The human placenta is a vascular rich tissue comprising mostly of foetal vessels ranging from large chorionic plate arteries and veins through to small resistance vessels and capillaries in the chorionic villus trees. (b) The umbilical cord contains one vein and two arteries which enter the surface of the placenta at the cord insertion and branch across the chorionic plate, providing numerous accessible arteries and veins of varying diameters.

Future advances in the development of in-vitro thrombosis models have the potential to move towards using human blood vessels perfused with human blood *ex vivo*. Human placental tissue represents a continuous and easily accessible source of human blood vessels, with arteries and veins ranging in size from large conduit vessels to small resistance vessels [64] (Fig. 2). There are 140 million live births globally each year [65] and most placentas are incinerated as clinical waste. The extended duration of viability of placental vessels makes them an appealing and feasible option for *ex vivo* studies, with most Universities associated with hospitals or located in proximity. Experimental studies have previously demonstrated perfusion of placental vessels for up to 6 h after delivery [66–68] with vessels remaining viable. It has also been demonstrated that vessels remain vasoactive >4 h after delivery, with nitric oxide still being produced [67], and endothelial cells remaining intact even in the absence of perfusion [69].

The accessibility of chorionic plate placental vessels (Fig. 2) facilities both in situ and isolated perfusion of vessels and makes them suitable for the same injury protocols used *in vivo* (e.g. laser injury or ferric chloride), offering the prospect of a bona fide human thrombosis model with the potential to replace animal experiments. The availability of numerous arteries and veins from the same placenta also allows simultaneous haemostasis assays to be performed in place of murine tail bleeding assays. Puncture wounds of a set diameter can be initiated using specific gauge needles [70] and the time to bleeding cessation or amount of blood loss can be measured.

As with any model, there are limitations with using placental vessels, these include immaturity in cell phenotype as the placental vessels are foetal origin. Our own work however has demonstrated that placental endothelial cells express similar levels of platelet regulators eNOS, COX1 and CD39 to HCAECs and protect against platelet thrombus formation to a similar extent when compared in microfluidic chambers (Riley *et al.*, unpublished data). As with most in-vivo models, the vessels will also be devoid of cardiovascular disease. However, there is the prospect of utilising placental vessels from pregnancies complicated by hypertension or diabetes to better model disease or using inflammatory mediators to evoke endothelial dysfunction. A benefit over in-vivo models however is the ability to treat or manipulate different components of the model in isolation. This is an exciting and revolutionary area of research, which requires future investigations to reveal the full potential of using human placental vessels *ex vivo*, as a robust, reproducible, and standardized method for investigating thrombosis and haemostasis.

## CONCLUSION

Significant developments have been made over the past few years adapting the standard in-vitro thrombosis model to enhance throughput and advance towards more complex microfluidic devices which better represent the vascular environment of arterial thrombosis. It is hoped that with these improved humanized thrombosis models, translation of research to human disease will be enhanced and animal usage in thrombosis research reduced.

## Acknowledgements

*None.*

### Financial support and sponsorship

*The work was supported by a British Heart Foundation/National Centre for the Replacement, Refinement and Reduction of Animals in Research studentship (NC/S001662/1) and a Biotechnology and Biological Sciences Research Council/ National Centre for the Replacement, Refinement and Reduction of Animals in Research project grant (NC/X002292/1).*

*Funding: BHF/NC3Rs joint studentship (NC/S001662/1); BBSRC/NC3Rs joint project grant (NC/X002292/1).*

### Conflicts of interest

*There are no conflicts of interest.*

## REFERENCES AND RECOMMENDED READING

Papers of particular interest, published within the annual period of review, have been highlighted as:

▪ of special interest
▪▪ of outstanding interest
